# Incidence and Risk Factors for Inappropriate Use of Non-Culture-Based Fungal Assays: Implication for Diagnostic Stewardship

**DOI:** 10.1093/ofid/ofab601

**Published:** 2021-12-06

**Authors:** Hiroshi Ito, Koh Okamoto, Shinya Yamamoto, Marie Yamashita, Yoshiaki Kanno, Daisuke Jubishi, Mahoko Ikeda, Sohei Harada, Shu Okugawa, Kyoji Moriya

**Affiliations:** 1 Department of Infectious Diseases, The University of Tokyo Hospital, Tokyo, Japan; 2 Division of Hospital Medicine, University of Tsukuba Hospital, Ibaraki, Japan; 3 Department of Infection Control and Prevention, The University of Tokyo Hospital, Tokyo, Japan

**Keywords:** beta-D glucan, galactomannan antigen, cryptococcal antigen, diagnostic stewardship

## Abstract

**Background:**

Non-culture-based fungal assays (NCBFAs) have been used increasingly to help diagnose invasive fungal diseases. However, little is known about inappropriate use of NCBFAs. We aimed to investigate inappropriate use of NCBFAs in a tertiary academic hospital.

**Methods:**

This retrospective cohort study included patients who underwent testing with beta-D glucan (BDG) between January and March 2018 or with galactomannan antigen (GMA) or cryptococcal antigen (CRAG) between January and June 2018. Testing was deemed appropriate if the clinical presentation was compatible with a fungal infection and there was a predisposing host factor at the time of ordering. We compared patients with appropriate and inappropriate use of NCBFAs using multivariate logistic regression analysis.

**Results:**

Four hundred seventy patients (BDG, 394; GMA, 138; CRAG, 164) met inclusion criteria and were evaluated. About 80% of NCBFAs were deemed inappropriate. Ordering by transplant medicine physicians, repetitions of the test, the absence of predisposing factors for fungal infections, and the absence of recommendations from infectious diseases consultants were associated with an increased risk of inappropriate NCBFA use.

**Conclusions:**

We found that a large proportion of NCBFAs were deemed inappropriate. There is an opportunity for diagnostic stewardship to reduce avoidable fungal testing among patients at low risk for fungal infection.

Invasive fungal diseases (IFDs) are clinically significant problems associated with high morbidity and mortality, particularly among immunocompromised patients [1]. Histopathologic and culture-based methods are the gold standard for diagnosing IFDs, but physicians often are faced with low-sensitivity methods, which may compromise the timely and accurate diagnosis of fungal infections [2, 3]. In view of this, non-culture-based fungal assays (NCBFAs) have been developed to help early diagnosis of IFDs. Among NCBFAs, beta-D glucan (BDG), galactomannan antigen (GMA), and cryptococcal antigen (CRAG) have been widely used to diagnose *Pneumocystis* pneumonia, candidiasis, aspergillosis, or cryptococcosis. They have also been shown to reduce inappropriate fungal treatment when negative results lower the possibility of IFDs [4].

While NBFAs are helpful, they have to be performed for those who benefit from them. Any diagnostic tests, especially if used in a low–pretest probability setting, could yield false positives or results of unknown clinical significance, leading to unwanted cascades (additional unnecessary tests or treatments and physical, psychological, and financial burden on patients) [5]. The significance of the inappropriate use of diagnostic methods has been increasingly recognized in the field of infectious diseases, such as *Clostridioides difficile* infections and urinary tract infections [6–11]. With respect to fungal testing, the lack of specificity of NCBFAs may drive unnecessary antifungal use despite their promising role in the diagnosis of IFDs [12]. For example, Fabre et al. reported an experience from an academic hospital in the United States where about half of the BDG orders were deemed inappropriate [13]. Besides, Szyszkowitz et al. reported the limited value of positive BDG tests during and after laparoscopic and open intestinal surgery [14]. However, data on the appropriateness of NCBFA use are still very limited.

Here we assess the clinical context and indications of NCBFAs to identify opportunities to improve practices and guide stewardship efforts at a tertiary academic hospital in Tokyo, Japan, serving large immunocompromised patient groups.

## METHODS

### Setting

The University of Tokyo Hospital is a large, single-site, tertiary teaching hospital with residents, fellows, and other trainees, located in the East of Japan with about 1200 beds, 360_ _000 inpatient admissions, and 700_ _000 total visits per annum. The hospital offers several specialist services, including solid organ transplantation (heart, lung, liver, and renal transplants), hematopoietic stem cell transplantation, neurosurgery, and pediatric surgery. The hospital does not have institutional guidelines or clinical pathways defining the indication of NCBFAs. The training physicians can place orders for testing without attending physicians’ instructions.

### Study Design and Patients

We conducted a retrospective cohort study of patients who underwent NCBFAs, including BDG, GMA, and CRAG tests, to clarify the frequency of inappropriate NCBFA orders and the factors predisposing inappropriate NCBFA orders. Approval from the institutional review board was obtained at the University of Tokyo Hospital (#11847). Because no care interventions were required, the requirement for signed patient consent was waived.

Patients who underwent either BDG, GMA, or CRAG tests during their hospital stay were included in this study. BDG, GMA, or CRAG can be ordered by any treating physician without oversight by the hospital stewardship team or infectious diseases consultants. Outpatients were excluded because clinical information such as vital signs, symptoms, and clinical course between hospital visits was poorly documented. Patients who had undergone hematopoietic stem cell transplant or solid organ transplant were also excluded because many of them received immunosuppressants, were at a high risk of fungal infections, and underwent protocol-driven serial surveillance with the combination of BDG, GMA, and CRAG. Of note, physicians (ie, liver surgeons) caring for transplant recipients often also cared for nontransplant patients (ie, patients with biliary tract malignancy) as well. Therefore, some tests included in this study were ordered by those physicians. To balance the number of tests analyzed, BDG tests for 3 months (January–March 2018) and GMA and CRAG tests for 6 months (January–June 2018) were examined because BDG tests were performed in-house with shorter turnaround times and were ordered much more often than the other 2. When a patient underwent the same NCBFA repeatedly, we only evaluated the clinical context and indications of the first.

### Outcomes

The primary outcomes were the frequency of inappropriate NCBFA orders among all NCBFA orders and the factors associated with inappropriate NCBFA orders. The number of the NCBFA order clusters (simultaneous order of BDG, GMA, and CRAG) and repetitions (repeated order of the same NCBFA) between January and June 2018 were also evaluated. The medical costs of inappropriate NCBFAs and subsequent tests were calculated in accordance with a fee-for-service system set by the Ministry of Health, Labor, and Welfare in Japan. The currency exchange rate used was US$1 to ¥100.

### Definitions

**Figure 1.**
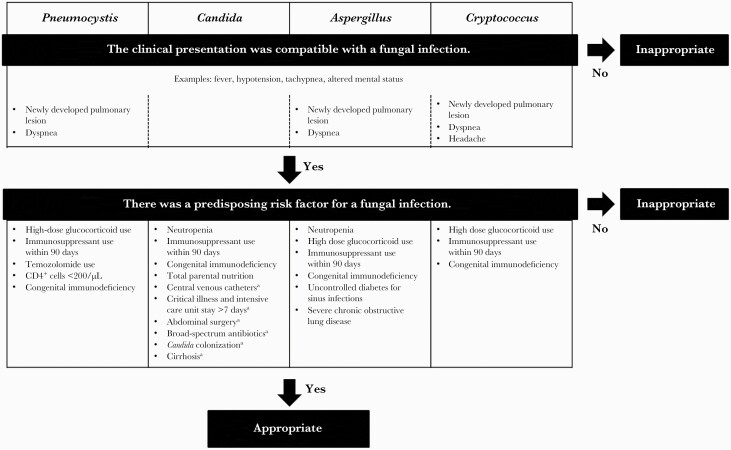
The criteria for determining appropriate and inappropriate testing. ^a^More than 1 category was needed for the patient to be considered at risk for invasive candidiasis.

Testing was deemed appropriate if there was a newly developed symptom or finding documented in medical records at the time of testing such as fever, dyspnea, newly identified pulmonary nodule, headache, or altered mental status (clinical criteria) AND there was a predisposing host factor (Figure 1) at the time of NCBFA ordering, according to Fabre et al. [13] Positive NCBFA results are defined as BDG >11 pg/mL (Wako, Tokyo, Japan), GMA index >0.5 (Bio-Rad Laboratories, Tokyo, Japan), and CRAG positive (Eiken, Tokyo, Japan) according to the instructions of each manufacturer.

### Variables

Patient medical records were manually reviewed to obtain the following variables: date of the tests, repetitions of the test in the same patient during the study period, simultaneous orders of other NCBFAs, presence of recommendations from infectious diseases (ID) consultants, patient age and sex, specialty of ordering physicians, stay in the intensive care unit, medical history (inherited severe immunodeficiency, asplenia, chronic obstructive pulmonary disease, sinus infection, chronic kidney disease on dialysis, chronic hepatitis C virus infection, cirrhosis, rheumatic condition, diabetes mellitus with hemoglobin A1c >7.0%, active solid organ tumor, and active hematologic malignancy), CD4-positive T-lymphocyte counts, neutrophil counts, receipt of immunosuppressant medications (glucocorticoids, biological agents, cyclosporine, tacrolimus, mycophenolate mofetil, and azathioprine), chemotherapy, and total parental nutrition, *Candida* colonization, documented bacteremia, and previous abdominal surgery. Specialty of ordering physicians was classified according to whether they engage in transplant medicine (transplant surgery, hematology, and pediatric hematology). Additional testing and treatment as a consequence of inappropriate NCBFAs were also reviewed.

### Statistical Analysis

Comparison between patients with appropriate NCBFAs and those with inappropriate NCBFAs was performed using the Fisher exact test for categorical variables and Mann-Whitney *U* test for continuous variables. We developed a multivariate logistic regression model, using the Akaike information criterion, to identify factors associated with appropriate NCBFAs. All variables were assessed for co-linearity, and interaction terms were tested. Results were presented as odds ratios and 95% CIs. *P* values <.05 were considered statistically significant. All statistical analyses were performed with EZR statistical software, version 1.41 [15].

## RESULTS

**Figure 2.**
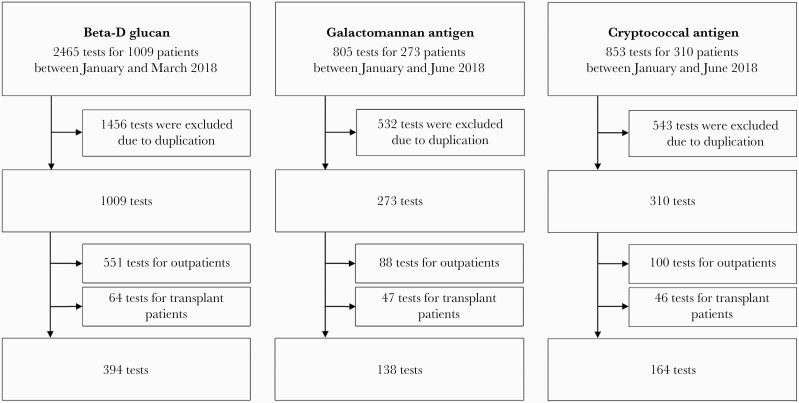
Selection of study subjects.

A total of 1159 patients underwent either serum BDG, GMA, or CRAG testing during the study period. After excluding 57 post-transplant inpatients and 632 outpatients, 470 patients (394 BDG tests, 138 GMA tests, and 164 CRAG tests) were included (Figure 2). The demographics of the study population are summarized in Table 1. Four hundred thirty-eight patients (93.2%) were aged 18 or older, and 241 patients (51.3%) were male. About two-thirds of included patients had either rheumatic diseases, solid tumors, or hematologic malignancies, and about half received immunosuppressants including glucocorticoid or chemotherapies. Notably, only 2 patients had microbiologically confirmed IFDs, both of which were proven candidemia. BDG tests for these patients were negative and deemed inappropriate by our definition as these patients had only 1 risk factor for invasive candidiasis (previous abdominal surgery). In addition, 2 patients had probable invasive aspergillosis. BDG and GMA tests for these patients were positive and deemed appropriate. No patients had known *Aspergillus* colonization before NCBFAs. No NCBFAs were ordered to monitor response to treatment.

**Table 1.** Clinical Characteristics of the Study Patients

	BDG (n = 394)	GMA (n = 138)	CRAG (n = 164)
Median age (IQR), y	64 (44–74)	51 (34–68)	52.5 (31–69)
Male	204 (51.8)	65 (47.1)	79 (48.2)
Malignancy	142 (36.0)	33 (23.9)	41 (12.8)
Solid malignancy	49 (12.4)	17 (12.3)	21 (12.8)
Hematologic malignancy	93 (23.6)	16 (11.6)	20 (12.2)
Congenital immunodeficiency	0 (0.0)	2 (1.4)	3 (1.8)
Chronic obstructive pulmonary disease	14 (3.6)	5 (3.6)	5 (3.0)
Sinusitis	49 (12.4)	17 (12.3)	21 (12.8)
Dialysis	18 (4.6)	4 (2.9)	6 (3.7)
Chronic HCV infection	4 (1.0)	2 (1.4)	2 (1.2)
Cirrhosis	23 (5.8)	28 (20.3)	29 (17.7)
Rheumatoid disease	96 (24.4)	46 (33.3)	54 (32.9)
Prior abdominal surgery	63 (16.0)	26 (18.8)	33 (20.1)
Asplenia	2 (0.5)	0 (0.0)	1 (0.6)
Neutropenia	20 (5.1)	9 (6.5)	10 (6.1)
HIV infection or AIDS	0 (0.0)	0 (0.0)	0 (0.0)
Diabetes mellitus with hemoglobin A1c >7.0%	30 (7.6)	6 (4.3)	10 (6.1)
Chemotherapy	96 (24.4)	26 (18.8)	29 (17.7)
High dose glucocorticoid	39 (9.9)	27 (19.6)	30 (18.3)
Immunosuppressant within 90 days	70 (17.8)	26 (18.8)	30 (18.3)
Total parenteral nutrition	32 (8.1)	6 (4.3)	8 (4.9)
Antibiotics use	205 (52.0)	72 (52.2)	86 (52.4)
Beta-lactams	98 (24.9)	40 (29.0)	47 (28.7)
Beta-lactams plus MNZ or CLDM	3 (0.8)	4 (2.9)	4 (2.4)
Vancomycin or teicoplanin	20 (5.1)	5 (3.6)	6 (3.7)
Trimethoprim-sulfamethoxazole	94 (23.9)	38 (27.5)	43 (26.2)
*Candida* from sputum	18 (4.6)	5 (3.6)	7 (4.3)
*Candida* from urine	2 (0.5)	3 (2.2)	3 (1.8)
Proven invasive fungal infection	1 (0.3)	1 (0.7)	1 (0.6)
Candidemia	1 (0.3)	1 (0.7)	1 (0.6)
Invasive pulmonary aspergillosis	0 (0.0)	0 (0.0)	0 (0.0)
Mucormycosis	0 (0.0)	0 (0.0)	0 (0.0)
Cryptococcosis	0 (0.0)	0 (0.0)	0 (0.0)
*Pneumocystis* pneumonia	0 (0.0)	0 (0.0)	0 (0.0)

Data are presented as No. (%) unless otherwise indicated.

Abbreviations: BDG, beta-D glucan; CLDM, clindamycin; CRAG, cryptococcal antigen; GMA, galactomannan antigen; HCV, hepatitis C virus; IQR, interquartile range; MNZ, metronidazole.

In total, about 80% of NCBFAs (BDG, 334 patients [74.8%]; GMA, 117 patients [74.8%]; CRAG, 146 patients [89.0%]) were deemed inappropriate. The result of the univariate analysis is shown in Table 2. BDG testing without predisposing factors for *Pneumocystis* pneumonia, candidiasis, and aspergillosis accounted for 65.9% of the inappropriate BDG testing. GMA and CRAG testing without a predisposing factor for aspergillosis and cryptococcosis infection accounted for 66.7% of the inappropriate GMA testing and 74.0% of the inappropriate CRAG testing, respectively. As for clinician factors, ordering by transplant medicine physicians was associated with inappropriate BDG orders, and the absence of recommendations from ID consultants was associated with inappropriate GMA and CRAG orders. Table 3 shows the results of multivariate logistic regression analysis for factors associated with appropriate NCBFAs. Ordering by transplant medicine physicians, repetitions of the test, the absence of predisposing factors for fungal infections, and the absence of recommendations from ID consultants were associated with an increased risk of inappropriate NCBFA use.

**Table 2.** Univariate Analysis of Factors Associated With Appropriate Testing of Non-Culture-Based Fungal Assay

	BDG (n = 394)			GMA (n = 138)			CRAG (n = 164)		
	Appropriate (n = 60)	Inappropriate (n = 334)	*P* Value	Appropriate (n = 21)	Inappropriate (n = 117)	*P* Value	Appropriate (n = 18)	Inappropriate (n = 146)	*P* Value
Median age (IQR), y	67.5 (45–75)	63 (44–73)	.25	40 (9–68)	51 (39–67)	.07	49.5 (29–62)	52.5 (35.5–70)	.27
Male	38 (63.3)	166 (49.7)	.068	12 (57.1)	53 (45.3)	.35	10 (55.6)	69 (47.3)	.62
Transplant medicine	16 (26.7)	136 (40.7)	<.005	11 (52.4)	40 (34.2)	.14	5 (27.8)	50 (34.2)	.79
Order repetition ≥2 times	31 (51.7)	168 (50.3)	.89	6 (28.6)	39 (33.3)	.8	6 (33.3)	42 (28.8)	.78
Simultaneous order of 3 NCBFAs	13 (21.7)	66 (19.8)	.73	11 (52.4)	68 (58.1)	.64	9 (50.0)	70 (47.9)	1
Recommendation from ID consultants	0 (0.0)	4 (1.2)	1	4 (19.0)	1 (0.9)	<.005	5 (27.8)	4 (2.7)	<.001
Solid malignancy	9 (15.0)	40 (12.0)	.53	3 (14.3)	14 (12.0)	.72	1 (5.6)	20 (13.7)	.47
Hematologic malignancy	15 (25.0)	78 (23.4)	.74	7 (33.3)	9 (7.7)	<.005	2 (11.1)	18 (12.3)	1
Congenital immunodeficiency	0 (0.0)	0 (0.0)	1	2 (9.5)	0 (0.0)	<.05	3 (16.7)	0 (0.0)	<.005
Chronic obstructive pulmonary disease	5 (8.3)	9 (2.7)	<.005	2 (9.5)	3 (2.6)	.17	1 (5.6)	4 (2.7)	.45
Sinusitis	2 (3.3)	8 (2.4)	.65	0 (0.0)	2 (1.7)	1	0 (0.0)	2 (1.4)	1
Dialysis	2 (3.3)	16 (4.8)	1	0 (0.0)	4 (3.4)	1	1 (5.6)	5 (3.4)	.51
Chronic HCV infection	1 (1.7)	3 (0.9)	.49	0 (0.0)	2 (1.7)	1	0 (0.0)	2 (1.4)	1
Cirrhosis	3 (5.0)	20 (6.0)	1	1 (4.8)	27 (23.1)	.075	2 (11.1)	27 (18.5)	.74
Rheumatoid disease	16 (26.7)	80 (24.0)	.63	6 (28.6)	40 (34.2)	.8	7 (38.9)	47 (32.2)	.6
Prior abdominal surgery	12 (20.0)	51 (15.3)	.34	4 (19.0)	22 (18.8)	1	2 (11.1)	31 (21.2)	.53
Asplenia	0 (0.0)	2 (0.6)	1	0 (0.0)	0 (0.0)	1	0 (0.0)	1 (0.7)	1
Neutropenia	14 (23.3)	6 (1.8)	<.0001	8 (38.1)	1 (0.9)	<.0001	3 (16.7)	7 (4.8)	.082
Diabetes mellitus with HbA1c >7.0%	3 (5.0)	27 (8.1)	.6	1 (4.8)	5 (4.3)	1	2 (11.1)	8 (5.5)	.3
Chemotherapy	18 (30.0)	78 (23.4)	.33	11 (52.4)	15 (12.8)	<.0005	5 (27.8)	24 (16.4)	.32
High-dose glucocorticoid	10 (16.7)	29 (8.7)	.063	8 (38.1)	19 (16.2)	<.01	8 (44.4)	22 (15.1)	<.01
Immunosuppressant within 90 d	16 (26.7)	54 (16.2)	.065	7 (33.3)	19 (16.2)	.075	7 (38.9)	23 (15.8)	<.05
Total parenteral nutrition	27 (45.0)	5 (1.5)	<.0001	3 (14.3)	3 (2.6)	<.05	2 (11.1)	6 (4.1)	.21
*Candida* from sputum	4 (6.7)	14 (4.2)	.5	1 (4.8)	4 (3.4)	.57	0 (0.0)	7 (4.8)	1
*Candida* from urine	0 (0.0)	2 (0.6)	1	0 (0.0)	3 (2.6)	1	0 (0.0)	3 (2.1)	1

Data are presented as No. (%) unless otherwise indicated. Transplant medicine includes transplant surgery, hematology, and pediatric hematology. High-dose glucocorticoid was defined as ≥20 mg of prednisone equivalents daily for ≥4 weeks.

Abbreviations: BDG, beta-D glucan; CRAG, cryptococcal antigen; GMA, galactomannan antigen; HCV, hepatitis C virus; ID, infectious diseases; IQR, interquartile range; NCBFA, non-culture-based fungal assay.

**Table 3.** Multivariate Logistic Regression Analysis of Factors Associated With Appropriate Testing of Non-Culture-Based Fungal Assay

	BDG (n = 394)			GMA (n = 138)			CRAG (n = 164)		
	Odds Ratio	95% CI	*P* Value	Odds Ratio	95% CI	*P* Value	Odds Ratio	95%CI	*P* Value
Transplant medicine	6.80	1.61–28.8	<.01						
Order repetition ≥2 times				12.4	1.21–127.0	<.05			
Recommendation from ID consultants				2.0 × 10^-3^	3.0 × 10^-5^–0.09	<.005	0.02	1.0 × 10^-3^–0.24	<.005
Chronic obstructive pulmonary disease	0.10	0.02–0.48	<.05						
Sinusitis	0.15	0.03–0.90	<.05						
Cirrhosis	0.15	0.02–0.99	<.05						
Neutropenia	3.0 × 10^-3^	6.0 × 10^-4^–0.02	<.001	8.0 × 10^-4^	3.0 × 10^-5^–0.02	<.001	0.06	0.01–0.40	<.005
High-dose glucocorticoid	0.26	0.09–0.76	<.01	0.20	0.05–0.88	<.05	0.11	0.03–0.47	<.005
Immunosuppressant within 90 d				0.11	0.02–0.56	<.01			
Total parental nutrition	6.0 × 10^-3^	2.0 × 10^-3^–0.002	<.001						

Transplant medicine includes transplant surgery, hematology, and pediatric hematology. High-dose glucocorticoid was defined as ≥20 mg of prednisone equivalents daily for ≥4 weeks.

Abbreviations: BDG, beta-D glucan; CRAG, cryptococcal antigen; GMA, galactomannan antigen; ID, infectious diseases.

**Figure 3.**
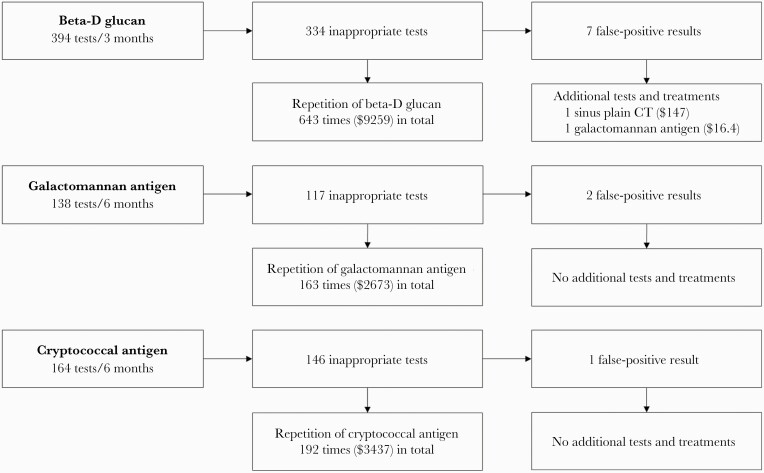
The consequence of inappropriate non-culture-based fungal assay. Abbreviation: CT, computed tomography.

Seventy-nine patients (16.8%) underwent 3 inappropriate NCBFAs simultaneously. The proportion of inappropriate testing was high whether there were single or simultaneous orderings. Furthermore, among 597 inappropriate NCBFA orders, 249 (41.7%) were followed by repeated tests (998 in total) during the study period. For inappropriate NCBFA orders (177 [29.6%]), tests were repeated ≥3 times. In total, 643 BDG, 163 GMA, and 192 CRAG tests, whether appropriate or not, were performed during the study period after the initial tests were deemed inappropriate (Figure 3). The medical cost of 1 BDG, 1 GMA, and 1 CRAG test in Japan is $14.4, $16.4, and $17.9, respectively. Thus, the estimated annual excess costs of repeated NCBFAs during the study period in patients with inappropriate NCBFAs were calculated as $18_ _518 for BDG, $10 682 for GMA, and $13_ _748 for CRAG. Furthermore, 10 patients (BDG, 7 patients; GMA, 2 patients; CRAG, 1 patient) had positive results that were considered false positive. Among those, 1 patient underwent additional sinus computed tomography, and none received antifungal treatment in response.

## DISCUSSION

In this study, we found that ~80% of the NCBFAs for hospitalized patients may not have been indicated and were therefore avoidable. The specialty of the physician who ordered the test, the risk factors for each IFD, and recommendation from an ID consultant were associated with increased appropriateness of NCBFA orders. A large proportion of patients who underwent inappropriate NCBFAs also underwent simultaneous and repeated NCBFAs.

In contrast to tests for *C. difficile* or urine culture, much less attention has been paid to NCBFAs. The study by Fabre and colleagues was the very first study on this topic and found that approximately half of BDGs were ordered inappropriately; the rate was higher in our study [13]. This difference might be explained by excessive surveillance testing for asymptomatic patients. Despite the difference, the high proportion of inappropriate BDG ordering is eye opening and calls for a better strategy to optimize BDG use. Our study extends the work by Fabre and colleagues by examining other NFBCAs (GMA and CRAG) and dissecting factors related to detailed patient background and ordering physicians.

In our study, physicians who care for transplant recipients were more likely than others to order NCBFAs inappropriately. We hypothesized that transplant physicians may have a practice habit of screening for fungal infections among transplant patients that they extend to nontransplant patients as a possible explanation for why they were more likely to order avoidable tests in these lower-risk patients. Inappropriate test ordering by specific clinical services has been reported previously; for example, a single-center retrospective study from Vanderbilt University Medical Center showed that duplicate type and screen tests for nontransplant patients were more common among adult services than pediatric services [16]. Additionally, several departments, especially where they care for transplant recipients, used NCBFAs in the routine order sets on admission or when working up for fever or increased inflammatory markers. However, we were unable to investigate what proportion of orders was part of order sets because our electronic medical record did not allow us to retrospectively determine it. Nonetheless, we speculate that its proportion would be close to that of simultaneous ordering of NCBFAs (16.8% in this study).

We found that inappropriate NCBFA ordering was common among non-ID physicians, especially when patients did not receive immunosuppressants and when they did not have signs and symptoms consistent with infectious diseases. In addition, we found that, as expected, recommendations from ID consultants were associated with appropriate use of NCBFAs. Multiple studies have shown that management led by ID consultants improves various outcomes in *Staphylococcus aureus* bacteremia [17, 18] and candidemia [19, 20], as well as antimicrobial stewardship [21]. Our findings add the promotion of appropriate diagnostic testing to the existing list of areas where the input of ID consultants can make an importance difference.

A large proportion of patients in this study underwent several NCBFAs simultaneously and repeated NCBFAs. Certainly, there are clinical situations where simultaneous ordering of multiple NCBFAs is appropriate and indeed required. BDG and GMA might be ordered for suspected aspergillosis. Ordering BDG, GMA, and CRAG at the same time may be needed if an immunocompromised patient presents with nonspecific lung infiltrates. However, if a patient with recent abdominal surgery develops fever on broad-spectrum antibiotics while receiving total parenteral nutrition through a central venous catheter, GMA and CRAG tests are likely inappropriate [22–25]. Furthermore, although we were unable to confirm that all of the repeated NCBFAs were inappropriate, inappropriate NCBFAs were often followed by many repeated NCBFA orders, which were very likely inappropriate as well. This may reflect a knee-jerk use of NCBFA orders, in which NCBFAs were ordered regularly regardless of the patient’s condition. Indeed, we observed that several departments included NCBFAs in routine order sets on admission or workup for fever or increased inflammatory markers.

Our study included predisposing factors for each IFD in the univariate and multivariate analyses. As these predisposing factors were included in the criteria for NCBFA appropriateness, it seems natural that these predisposing factors were associated with appropriate NCBFA orders. However, some of the predisposing factors for IFDs, such as abdominal surgery, *Candida* colonization, and uncontrolled diabetes, were not associated with appropriate NCBFA orders. This suggests that some of these factors may not be recognized as predisposing factors for IFDs by non-ID physicians.

Inappropriate ordering and frequent simultaneous and repeated ordering of NCBFAs may reflect non-ID physicians’ lack of knowledge on fungal infections and the differences among them. This problem is not unique to fungal diagnostics. It has been reported that general practitioners who are unfamiliar with HIV infection tend to order inappropriate tests [26]. A single-center retrospective study from the University of Washington showed that 45% of specialized laboratory tests for patients with HIV infection were not indicated. Educational interventions [27, 28], modification of electronic ordering systems [29, 30], and display of test price [31] have been attempted to reduce inappropriate testing, and these interventions have potential for application in the field of fungal diagnostic stewardship. Our findings suggest that stewardship efforts engaging transplant physicians, clinical decision support tools to guide ordering practices, and involvement of ID physicians as stewards may be important strategies to improve fungal testing practices.

Several limitations of this study should be acknowledged. First, there is no clearly and widely accepted consensus on appropriateness of NCBFAs. The criteria we used for the study might not accurately capture inappropriate cases, although the predisposing factors in our criteria are largely consistent with the recently published consensus definitions of IFDs from the European Organization for Research and Treatment of Cancer and the Mycoses Study Group [23]. Second, it was a single-center, retrospective study in a tertiary care academic center in Japan, where physicians offer solid organ and hematopoietic stem cell transplantation. Therefore, the results may not be generalizable to other institutions with different patient, physician, and health care characteristics as well as ordering cultures. Although we attempted to ascertain the classification and associated factors with meticulous manual chart review, the results could have been subject to incomplete documentation. Third, there were only 2 patients with confirmed IFDs, thus precluding the application of the results to populations with higher incidences of IFDs but supporting the finding of excessive ordering. Fourth, we did not assess the appropriateness of subsequent NCBFAs for those who ordered repeatedly in the same patients, and these tests may have been appropriate. Lastly, we did not fully explore the drivers of inappropriate testing. The problem is likely multifactorial, with misuse of order sets and lack of knowledge on fungal diagnostics among non-ID physicians being the most important drivers. A future survey or qualitative analysis to elucidate the drivers in depth is needed to guide interventions including education.

In conclusion, we found that a large proportion of NCBFAs were ordered inappropriately. The absence of predisposing factors, the specialty of the physicians, and the absence of recommendations from ID consultants were associated with the inappropriate NCBFAs. Fungal diagnostic stewardship, such as guidance on patient selection for NCBFAs, is needed to reduce the inappropriate ordering of fungal testing and to prevent cascades of unnecessary care.

## References

[1] Falci DR , Stadnik CMB, Pasqualotto AC. A review of diagnostic methods for invasive fungal diseases: challenges and perspectives. Infect Dis Ther 2017; 6:213–23. 28357708 10.1007/s40121-017-0154-1 PMC5446367

[2] Clancy CJ , Nguyen MH. Finding the “missing 50%” of invasive candidiasis: how nonculture diagnostics will improve understanding of disease spectrum and transform patient care. Clin Infect Dis 2013; 56:1284–92. 23315320 10.1093/cid/cit006

[3] Patterson TF , Thompson GR 3rd, Denning DW, et al. Practice guidelines for the diagnosis and management of aspergillosis: 2016 update by the Infectious Diseases Society of America. Clin Infect Dis 2016; 63:e1–60. 27365388 10.1093/cid/ciw326 PMC4967602

[4] Ito-Takeichi S , Niwa T, Fujibayashi A, et al. The impact of implementing an antifungal stewardship with monitoring of 1-3, β-D-glucan values on antifungal consumption and clinical outcomes. J Clin Pharm Ther 2019; 44:454–62. 30723924 10.1111/jcpt.12809

[5] Ganguli I , Simpkin AL, Lupo C, et al. Cascades of care after incidental findings in a US national survey of physicians. JAMA Netw Open 2019; 2:e1913325. 31617925 10.1001/jamanetworkopen.2019.13325 PMC6806665

[6] Rock C , Pana Z, Leekha S, et al. National Healthcare Safety Network Laboratory-identified *Clostridium difficile* event reporting: a need for diagnostic stewardship. Am J Infect Control 2018; 46:456–8. 29305285 10.1016/j.ajic.2017.10.011 PMC6734925

[7] Friedland AE , Brown S, Glick DR, et al. Use of computerized clinical decision support for diagnostic stewardship in *Clostridioides difficile* testing: an academic hospital quasi-experimental study. J Gen Intern Med 2019; 34:31–2. 30218259 10.1007/s11606-018-4659-4 PMC6318163

[8] Yen C , Holtom P, Butler-Wu SM, et al. Reducing *Clostridium difficile* colitis rates via cost-saving diagnostic stewardship. Infect Control Hosp Epidemiol 2018; 39:734–6. 29611494 10.1017/ice.2018.51

[9] Sullivan KV , Morgan DJ, Leekha S. Use of diagnostic stewardship practices to improve urine culturing among SHEA Research Network hospitals. Infect Control Hosp Epidemiol 2019; 40:228–31. 30522544 10.1017/ice.2018.325

[10] Claeys KC , Blanco N, Morgan DJ, et al. Advances and challenges in the diagnosis and treatment of urinary tract infections: the need for diagnostic stewardship. Curr Infect Dis Rep 2019; 21:11. 30834993 10.1007/s11908-019-0668-7

[11] Watson KJ , Trautner B, Russo H, et al. Using clinical decision support to improve urine culture diagnostic stewardship, antimicrobial stewardship, and financial cost: a multicenter experience. Infect Control Hosp Epidemiol 2020; 41:564–70. 32131910 10.1017/ice.2020.37

[12] Alegria W , Patel PK. The current state of antifungal stewardship in immunocompromised populations. J Fungi 2021; 7:352. 10.3390/jof7050352 PMC8145600 33946217

[13] Fabre V , Markou T, DeMallie K, et al. Single academic center experience of unrestricted β-d-glucan implementation. Open Forum Infect Dis 2018; 5:XXX–XX. 10.1093/ofid/ofy195 PMC6120669 30186888

[14] Szyszkowitz A , Zurl C, Herzeg A, et al. Serum 1,3-beta-D-glucan values during and after laparoscopic and open intestinal surgery. Open Forum Infect Dis 2018; 5:XXX–XX. 10.1093/ofid/ofy296 PMC6290064 30568978

[15] Kanda Y. Investigation of the freely available easy-to-use software “EZR” for medical statistics. Bone Marrow Transplant 2013; 48:452–8. 23208313 10.1038/bmt.2012.244 PMC3590441

[16] Compton ML , Szklarski PC, Booth GS. Duplicate type and screen testing: waste in the clinical laboratory. Arch Pathol Lab Med 2018; 142:358–63. 29210591 10.5858/arpa.2016-0629-OA

[17] Vogel M , Schmitz RP, Hagel S, et al. Infectious disease consultation for *Staphylococcus aureus* bacteremia - a systematic review and meta-analysis. J Infect 2016; 72:19–28. 26453841 10.1016/j.jinf.2015.09.037

[18] Forsblom E , Frilander H, Ruotsalainen E, Järvinen A. Formal infectious diseases specialist consultation improves long-term outcome of methicillin-sensitive *Staphylococcus aureus* bacteremia. Open Forum Infect Dis 2019; 6:XXX–XX. 10.1093/ofid/ofz495 PMC7047950 32128337

[19] Lee RA , Zurko JC, Camins BC, et al. Impact of infectious disease consultation on clinical management and mortality in patients with candidemia. Clin Infect Dis 2019; 68:1585–7. 30281081 10.1093/cid/ciy849

[20] Ishikane M , Hayakawa K, Kutsuna S, et al. The impact of infectious disease consultation in candidemia in a tertiary care hospital in Japan over 12 years. PLoS One 2019; 14:e0215996. 31022251 10.1371/journal.pone.0215996 PMC6483235

[21] Ostrowsky B , Banerjee R, Bonomo RA, et al. Infectious diseases physicians: leading the way in antimicrobial stewardship. Clin Infect Dis 2018; 66:995–1003. 29444247 10.1093/cid/cix1093

[22] Patterson KC , Strek ME. Diagnosis and treatment of pulmonary aspergillosis syndromes. Chest 2014; 146:1358–68. 25367472 10.1378/chest.14-0917

[23] Donnelly JP , Chen SC, Kauffman CA, et al. Revision and update of the consensus definitions of invasive fungal disease from the European Organization for Research and Treatment of Cancer and the Mycoses Study Group Education and Research Consortium. Clin Infect Dis 2020; 71:1367–76. 31802125 10.1093/cid/ciz1008 PMC7486838

[24] Lin YY , Shiau S, Fang CT. Risk factors for invasive *Cryptococcus neoformans* diseases: a case-control study. PLoS One 2015; 10:e0119090. 25747471 10.1371/journal.pone.0119090 PMC4352003

[25] Henao-Martínez AF , Gross L, Mcnair B, et al. Risk factors for cryptococcal meningitis: a single United States center experience. Mycopathologia 2016; 181:807–14. 27502502 10.1007/s11046-016-0048-x PMC5121069

[26] Bolles K , Woc-Colburn L, Hamill RJ, Hemmige V. Ordering patterns and costs of specialized laboratory testing by hospitalists and house staff in hospitalized patients with HIV at a county hospital: an opportunity for diagnostic stewardship. Open Forum Infect Dis 2019; 6:ofz158. 31205970 10.1093/ofid/ofz158 PMC6557192

[27] Miyakis S , Karamanof G, Liontos M, Mountokalakis TD. Factors contributing to inappropriate ordering of tests in an academic medical department and the effect of an educational feedback strategy. Postgrad Med J 2006; 82:823–9. 17148707 10.1136/pgmj.2006.049551 PMC2653931

[28] Morgan S , Morgan A, Kerr R, Tapley A, Magin P. Test ordering by GP trainees: effects of an educational intervention on attitudes and intended practice. Can Fam Physician 2016; 62:733–41. 27629671 PMC5023346

[29] Iturrate E , Jubelt L, Volpicelli F, Hochman K. Optimize your electronic medical record to increase value: reducing laboratory overutilization. Am J Med 2016; 129:215–20. 26475957 10.1016/j.amjmed.2015.09.009

[30] Sadowski BW , Lane AB, Wood SM, Robinson SL, Kim CH. High-value, cost-conscious care: iterative systems-based interventions to reduce unnecessary laboratory testing. Am J Med 2017; 130:1112.e1–7. 10.1016/j.amjmed.2017.02.029 28344140

[31] Yarbrough PM , Kukhareva PV, Horton D, Edholm K, Kawamoto K. Multifaceted intervention including education, rounding checklist implementation, cost feedback, and financial incentives reduces inpatient laboratory costs. J Hosp Med 2016; 11:348–54. 26843272 10.1002/jhm.2552

